# Foreign Correspondence

**Published:** 1839-02-23

**Authors:** R. Harlan


### FOREIGN CORRESPONDENCE.

To the Editors of the Medical Examiner.

*London*, January 15th, 1839.

Gentlemen,—I hope you will have received ere this arrives, a long letter* I had the honour to address to the Editors of the Medical Examiner. Since our last we have passed much of our time in the Royal College and British Museum—two exhaustless resources of amusement and instruction; and, in connexion with the practice and museums of the various hospitals and medical schools of this boundless metropolis, constituting a most valuable resort for the student or physician who has already finished a practical course of studies in the less expensive institutions of Paris. Agreeably to the appointment alluded to in our last, we waited on Sir Astley Cooper, to witness his interesting operation on the rabbit, in which, and in other small animals, he induces death in a very short space of time, by the simultaneous pressure on the carotid and vertebral arteries of both sides,—and, as he avers, absolutely without *pain* to the animal. He caused me to repeat the experiment before him, in order to assure himself that we adequately comprehended the modus operandi.

We then took our leaves, with the letters of introduction, which he had so obligingly offered us, to MM. Roux, Velpeau, Lisfranc, and Fisher, of Paris.

During our visit to the Royal College of Surgeons this morning, Mr. Owen afforded us a microscopical view of the new species of Entozoon, discovered by him in 1835; it exists, in incalculable numbers, in some men, women, and children, both in healthy and in diseased bodies,—the muscles appear studded over with white dots, as if disseminated with calcareous depositions,—and those most commonly affected are the great pectoral, latissimus dorsi, abdomiles, &c.; no muscles, however, being exempt. The worms have also been detected in Prussia, but not as yet in Paris. As Mr. Owen has already well described this parasite, under the name of "*Trichina spiralis*," in the Encyclopœdia of Anatomy and Physiology of Tod and others, I have only to add, that Mr. Owen kindly presented me with specimens, requesting me to ascertain if the same exist in Americans.

We have been exceedingly interested in attending the meetings of the various learned societies in London; in fact, every evening in the week might be thus advantageously spent. The Royal Society, the Linnæan, and Geological, meet once a fortnight, all on different nights, and a club dinner is given by each immediately preceding the meeting. We have found the fare excellent on these occasions, where good company, good wine, and good order prevail. We had a very interesting discussion at the Geological Society, based on a memoir which I read on the fossil remains of the immense extinct animal from Alabama, which I had described under the name of *Basilosaurus*,—the question to be determined being, whether or not this fossil belonged really to the Saurian or Mammiferous order. The strongest argument of its mammiferous nature, was derived from certain analogies which Mr. Owen discovered in his microscopical examinations of sections of the teeth of this fossil, and those of the *Dugoney*. For the detailed account of this discussion, I must refer you to the notice published in the Athenæum a few days subsequently, and to the forthcoming volume of the Transactions of the London Geological Society.

London is at present enlightened by hundreds of lecturers on all subjects. We listened to a very interesting clinical discourse to-day, by Sir Benjamin Brodie, at St. George's Hospital, on certain diseases of the bones.

Drs. Hall and Grainger are delivering admirable lectures on the nervous system, ably illustrated by comparative anatomy; and Dr. Grant's discourses on comparative anatomy, would in themselves compensate for a voyage across the Atlantic. We are to attend the Royal Geographical Society's meeting, on Wednesday, and to hear Professor Faraday lecture at the Royal Institution, on Friday evening next. On Sunday last, we again had the honour to meet at Mr. W. Lawrence's hospitable board, a very agreeable society of ladies and gentlemen.

Among the liberal dispensers of hospitality to scientific foreigners, and more particularly to their "*transatlantic brethren*,"—for this is the designation which, by common consent, is hereafter to be applied to our compatriots,—none surpass our enlightened friend, Mr. Lawrence; the high encomiums bestowed by this admirable writer on American institutions and our men of science, in his celebrated work, "The Physiology and Natural History of Man," together with the friendly disposition he has ever been so ready to show to members of our profession vi-

* This letter was probably in the mail-bag of one of the lost packets.—Eds.

siting London, has long rendered his name particularly popular with us. I am aware that his unsurpassed professional fame, either as a writer, lecturer, or practitioner of surgery, can receive no addition from any encomiums in my power to express, but in thus casually alluding to the subject at all, I could not say less of one, to whose liberal hospitality myself and numerous friends have so often and so extensively been indebted. His merits, indeed, could be only adequately expressed, by stating that they are equalled by the brilliant success which has finally crowned his intellectual and professional exertions. I presume that his income is surpassed by very few of his European competitors. The trustees of the Royal College of Surgeons have displayed their discrimination, and, at the same time, conferred a well-merited compliment on Mr. Lawrence, in chosing him as the principal director of that magnificent and unrivalled anatomical and physiological cabinet, which has grown out of Hunter's museum, a colossal monument of British taste and of British munificence, contemplated with sentiments of pride and delight by every true votary of science, and which is admirably calculated to perpetuate the fame and hand down the name of its illustrious founder and enlightened directors to the latest posterity.

I have been examining, with great interest, at the College of Surgeons, a descriptive catalogue of the museum, preparing by Messrs. Clift and Owen; several volumes in quarto have already been published, they are illustrated with numerous plates, and would form a most valuable acquisition to our public libraries and scientific institutions. I hope you will recommend the purchase of a copy for the library of the Philadelphia Hospital. I presume there could be no difficulty in the American Philosophical Society and Academy of Natural Sciences of Philadelphia making an exchange, pro rata, for this invaluable contribution to physiology and anatomy.

In going the rounds, with Mr. Mayo, at the Middlesex Hospital, he showed us some cases of considerable interest; among them, cases of "Acute Anchylosis" of the knee-joint, distinguishable at first from common cases ending in anchylosis, in the inflammation and pain being unattended with swelling of the joint, and the disorganization and stiffness being completed in two or three months. Sir Benjamin Brodie has also noticed the same variety of disease, and I think both these surgeons have published their experience in the recent number of the Medico-Chirurgical Transactions.

I had the pleasure of meeting Sir Benjamin Brodie, and twelve other eminent members of the profession, including Carswell, Grant, Sharpey, &c., at the house of Professor B. Phillips,—gentlemen, whose scientific acquirements are far from being limited to their own immediate professions. Sir B. B. considers it easy to distinguish scrofulous from syphilitic caries of the bones ab origine, by the latter affection being always preceded by evident symptoms of pain and inflammation; whilst the first indication of strumous caries, is a small, hard, indolent, osseous tumour in different parts of the frame, notably in the metatarsal and metacarpal bones; and even at this early period of the disease, it is already too late for the successful operation of any remedies; no means can prevent, sooner or later, suppuration of the neighbouring soft parts, and subsequent caries, which is not superficial, either, but is seated deep in the bone; hence Mr. B. says the disease must have originated in the medullary portion.

*Saturday*, January 19th.—Last evening we were delighted with Mr. Faraday's discourse, at the Royal Institution, on the physiology of the torpedo, gymnotics and silucus, as connected with electricity. He has recently been occupied in experimenting on a torpedo now living at the Gallery of Practical Science, Adelaide street. Mr. F. is a superlatively neat manipulator, and eloquent lecturer; he riveted the attention of a numerous and intellectual audience, including many distinguished foreigners, both of the scientific and diplomatic corps. We strike our tent, and embark for Havre to-morrow. I hope soon to communicate with you from Paris.

Respectfully, yours, etc.

R. Harlan.

